# Recent progress and current opinions in Brillouin microscopy for life science applications

**DOI:** 10.1007/s12551-020-00701-9

**Published:** 2020-05-26

**Authors:** Giuseppe Antonacci, Timon Beck, Alberto Bilenca, Jürgen Czarske, Kareem Elsayad, Jochen Guck, Kyoohyun Kim, Benedikt Krug, Francesca Palombo, Robert Prevedel, Giuliano Scarcelli

**Affiliations:** 1 Photonics Research Group, INTEC, Ghent University-imec, 9052 Ghent, Belgium; 2 Present Address: Present address: Dipartimento di Fisica, Politecnico di Milano, Piazza Leonardo da Vinci 32, I-20133 Milan, Italy; 3 Biotechnology Center, TU Dresden, Dresden, Germany; 4 Max Planck Institute for the Science of Light & Max-Planck-Zentrum für Physik und Medizin, Erlangen, Germany; 5 Biomedical Engineering Department, Ben-Gurion University of the Negev, Beersheba, Israel; 6 Laboratory of Measurement and Sensor System Technique, TU Dresden, Dresden, Germany; 7 Cluster of Excellence Physics of Life, TU Dresden, Dresden, Germany; 8 Advanced Microscopy, Vienna Biocenter Core Facilities (VBCF), Vienna, Austria; 9 School of Physics and Astronomy, University of Exeter, Exeter, UK; 10 Cell Biology and Biophysics Unit, European Molecular Biology Laboratory (EMBL), Heidelberg, Germany; 11 Fischell Department of Bioengineering, University of Maryland, College Park, MD USA

**Keywords:** Brillouin microscopy, Optical elastography, Biomechanics

## Abstract

Many important biological functions and processes are reflected in cell and tissue mechanical properties such as elasticity and viscosity. However, current techniques used for measuring these properties have major limitations, such as that they can often not measure inside intact cells and/or require physical contact—which cells can react to and change. Brillouin light scattering offers the ability to measure mechanical properties in a non-contact and label-free manner inside of objects with high spatial resolution using light, and hence has emerged as an attractive method during the past decade. This new approach, coined “Brillouin microscopy,” which integrates highly interdisciplinary concepts from physics, engineering, and mechanobiology, has led to a vibrant new community that has organized itself via a European funded (COST Action) network. Here we share our current assessment and opinion of the field, as emerged from a recent dedicated workshop. In particular, we discuss the prospects towards improved and more bio-compatible instrumentation, novel strategies to infer more accurate and quantitative mechanical measurements, as well as our current view on the biomechanical interpretation of the Brillouin spectra.

## Introduction

Brillouin light scattering spectroscopy is based on the interaction of light with traveling density fluctuations (acoustic waves or “phonons”), and since its discovery almost a century ago (Brillouin 1922) has become a widely used and highly appreciated standard technique for studying condensed matter systems (Dil 1982). The spectrum of the Brillouin scattered light gives access to the mechanical properties of the sample through the longitudinal modulus, *M*. Specifically, the measured so-called Brillouin frequency shift, *ν*_*B*_, is related to the medium’s sound velocity (*V*), via the relation *v*_*B*_ = 2*n*/*λV* sin (*θ*/2), where *n* is the material refractive index, λ is the incident wavelength, and *θ* is the angle between the incident and scattered light. The sound velocity is in turn directly related to the elastic (“storage”) modulus of the material through *M* = *V*/*ρ*^2^, where *ρ* is the mass density. On the other hand, the width *∆*_*B*_ of the Brillouin spectrum is dependent on the sample’s dissipative mechanical (viscous) properties (Dil 1982; Berne and Pecora 2000). Here, it is important to note that Brillouin scattering probes the material properties in the GHz frequency range, which is in contrast to many existing methods currently utilized in biomechanics. This, together with the definition of the longitudinal modulus, means that the elasticity measured by Brillouin scattering will typically assume much higher values (in the GPa range) for common (bio-) materials, compared with the widely used Young’s modulus *E* (often in the kPa range). Nevertheless, recent advances in spectrometer design have opened up the possibility of applying Brillouin spectroscopy to live biological systems (Scarcelli and Yun 2007; Scarcelli et al. 2015). This in turn has attracted increasing interest in the field of biomechanics, due to its unrivaled ability to map, and thus directly “see,” viscoelastic properties of living matter in 3D and in a non-contact, label-free and high-resolution fashion. This is especially the case when integrated with common scanning (e.g., confocal) microscopes (Palombo and Fioretto 2019; Elsayad et al. 2019; Prevedel et al. 2019). These advances have paved the way for the emerging field of “Brillouin microscopy,” which has already led to a wide range of applications in biology, allowing the investigation of mechanics from the (sub-)cellular (Scarcelli et al. 2015; Antonacci et al. 2018; Mattana et al. 2018; Zhang et al. n.d.) to the tissue (Elsayad et al. 2016; Schlussler et al. 2018; Bevilacqua et al. 2019) and whole organism scale (Pukhlyakova et al. 2018), as well as applications to medicine (e.g., (Scarcelli et al. 2012; Palombo et al. 2018; Steelman et al. 2015)). In this communication, we discuss recent progress and trends in Brillouin microscopy, with a focus on high-speed, yet biology compatible, instrumentation. We also touch upon some best practices for extracting accurate and quantitative results from measurements and share our current view on the biomechanical interpretation of the Brillouin spectra in complex heterogeneous systems (such as are characteristic for biological samples).

## Approaches and future prospects for high-performance instrumentation

Current physical approaches for implementing BM can broadly be divided into two categories, depending on whether the photons scatter off spontaneous or coherently driven phonons. The latter, which are also referred to as “stimulated” techniques (ST), can in turn be divided into two categories: stimulated Brillouin scattering (SBS) (Ballmann et al. 2015; Remer and Bilenca 2016; Remer et al. 2019), which currently employs tunable continuous-wave (cw) lasers, and impulsive stimulated Brillouin scattering (ISBS) (Ballmann et al. 2017; Krug et al. 2019), which is based on ultrashort pulse laser excitation. While most realizations to date utilize spontaneous scattering (e.g., (Scarcelli and Yun 2007; Scarponi et al. 2017)), recent technical developments in the field of SBS have sparked considerable interest and prompted our community to critically evaluate and discuss which of these approaches is the most promising one in terms of desirable acquisition speeds as well as spatial and spectral resolution, while maintaining sample viability. In ST, an interference fringe pattern is created within the sample via two overlapping laser (pump) beams which excites a standing acoustic wave through electrostriction. These stimulated “phonons” can then be probed by spatially overlapping a frequency-tunable “probe” laser beam. When the frequency detuning of these lasers is around the Brillouin shift of the sample light is scattered to/from the probe beam, resulting in stimulated Brillouin gain/loss. Here, it is worth noting that the active stimulation of phonons in ST can result in measured attenuation coefficients and thus linewidths which are different from those measured by spontaneous Brillouin scattering (Remer and Bilenca 2016; Krug et al. 2019). Nevertheless, stimulated techniques offer unprecedented and exciting advantages, such as a superior spectral resolution and access to the mass density—two properties that can in principle enable one to more accurately distinguish different biomechanical constituents within the measurement volume as well as to quantify the obtained spectra in terms of mechanical modulus. Hence, we believe these developments may provide future research avenues in biology and help establish ST as a complementary method to current approaches based on spontaneous Brillouin scattering. Current ST implementations (Remer et al. 2019; Krug et al. 2019) however still require high laser powers and a “transmission” geometry, i.e., simultaneous optical access to the probed region in a sample from opposite sides, which can sometimes prove undesirable. Though the high laser powers are somewhat mitigated by the use of Near Infra-red (NIR) laser sources (Remer et al. 2017), in current implementations these conditions can prove restrictive for studying non-transparent or photosensitive samples, or for studies over extended time periods. However, the absence of the elastic scattering signal (background) in ST can be particularly desirable, and the very high spectral resolution can in certain cases also prove desirable or essential for resolving subtle changes or features in the Brillouin scattering spectra or distinguishing closely spaced peaks in frequency. As such there are clear advantages as well as caveats to ST in their current implementations. Here, it is also worth noting that STs are at an earlier development stage than spontaneous ones, thus dramatic improvements in the near future can be expected. For example, current implementation of SBS uses cw lasers, and thus, their regime of operation does not fully take advantage of the non-linearity of the stimulated process, which is recently being exploited in pulsed-based variants. Future improvements, which have to come hand-in-hand with more advanced lasers, might come naturally—spontaneous BM has indeed benefited from more than a decade of focused method developments. Consequently, spontaneous BM is now a mature method that has proven to be valuable in many biomedical applications. A more quantitative assessment of the current state-of-the-art in regard to the performance of the different approaches is provided in Table 1.

**Table 1:** Current performance parameters for major variants of Brillouin microscopy implementations. All parameters were obtained on water samples, except (Krug et al. 2019), and from references or supplied and updated by the respective authors. *ISBS*, impulsive stimulated Brillouin scattering; *VIPA*, virtually imaged phase array; *TFPI*, tandem multi-pass Fabry-Pérot interferometer. Dwell time for ISBS represents effective measurement time for (Krug et al. 2019). Relative precision is defined as the ratio of the instrument’s precision to typical Brillouin shifts measured (e.g., water). Optical resolution refers to the extent of the optical measurement volume (point spread function, PSF). Linewidth fidelity refers to the ability and accuracy of estimating the “true” linewidth from the raw spectral data. For techniques with more than one reference listed, the quoted values are taken from the references marked “*”. Linewidth fidelity ranges from x=low to xxx=high for clarity

Technique	Spectral resolution (MHz)	Dwell time/pixel (ms)	Power at sample (mW)/effective NA	Precision (MHz)	Relative precision	Optical resolution (*x*/*z*, μm)	Linewidth fidelity
SBS (Remer and Bilenca 2016)* (Remer et al. 2019)** (Ballmann et al. 2015; Remer et al. 2017) 780 nm	~ 30–100	2–20	265/0.25–0.7	12	0.002	0.8 × 16*	xxx
						0.3 × 2**	
ISBS (Krug et al. 2019)* (Ballmann et al. 2017) 532/780/895 nm hydrogel	3–6	0.1	35/0.025	0.4	0.005	10 × 230	xxx
Confocal VIPA (Scarcelli et al. 2015)* (Elsayad et al. 2016; Bevilacqua et al. 2019; Zhang et al. 2017; Antonacci and Braakman 2016) 532 nm	600	50	11/0.6	10.0	0.001	0.25 × 0.7	xx
Confocal VIPA (Nikolic and Scarcelli 2019) 660 nm	500	20	61/0.95	8.0	0.002	0.5 × 2	xx
In vivo confocal VIPA (Schlussler et al. 2018; Besner et al. 2016)* 780 nm	600	200	2 /0.1	16.0	0.003	4 × 60	xx
Line-scan VIPA (Zhang et al. 2016) 532 nm	470	< 1	100/0.1	10	0.002	3.3 × 18	x
Confocal TFPI (Mattana et al. 2018)* (Caponi et al. 2001) 532 nm	~ 100	> 1000	< 3.5/1.2	< 10	0.001	0.5 × 8	xx

It is worth noting that, as with many optical measurement techniques, both spontaneous and stimulated BM are subject to common trade-offs in performance in terms of achievable spectral precision, spatial as well as temporal resolution and potential photodamage. In particular, in the case of considerable signal averaging (as for ISBS), it is important to realize that the measurement duration increases linearly with the number of averaged signals, but the SNR increases with the square-root of the number of averaged signals only. As such, when considering the representative values quoted in Table 1, it should be kept in mind that in many cases it is possible to e.g., increase the acquisition speed or spectral resolution, or reduce the laser intensity, at the expenses of the other parameters.

## Strategies for properly assessing and avoiding photodamage

Although Brillouin microscopy probes mechanical properties in a non-contact fashion using light, the weak scattering cross section often necessitates potentially harmful illumination intensities that can range from a few mW to several hundred of mW in some practical implementations. While BM of human tissues has previously been Institutional Review Board (IRB) approved for < 10 mW and is currently used in clinical trials to assess corneal biomechanics (Yun and Chernyak 2018; Shao et al. 2019), higher laser powers or more photosensitive samples raise concerns about phototoxic effects and concomitant alterations of biological or mechanical properties in the biological samples under investigation. Therefore, this calls for a general consensus on best practices for assessing and avoiding photodamage in Brillouin microscopy. In this direction, it is our current standpoint that better experimental design and more stringent controls are needed, as cellular mechanics are susceptible to changes on the sub-second time scale and therefore damage can occur during typical image acquisition times. In designing proper imaging experiments, it is important to critically consider the overall illumination dosage, i.e., intensity × time, which has been shown to be the main variable affecting phototoxicity (Icha et al. 2017). Furthermore, laser illumination wavelength plays a considerable role, with NIR illumination being better tolerated than the widely used 532 nm laser line. At present, it seems that currently available laser lines such as 660 nm (Nikolic and Scarcelli 2019) and 780 nm (Schlussler et al. 2018) provide an acceptable compromise between having a reduced Brillouin scattering cross section while benefitting from minimized risk of photodamage. In some sense, there are obvious parallels to the field of optical trapping as introduced by Arthur Ashkin in the 1970s. After first using 514 nm Argon ion lasers for trapping glass beads, when moving towards biological samples Ashkin soon realized that this wavelength leads to cell “opticution,” which was later avoided by resorting to 1060 nm Nd:YAG lasers (Ashkin et al. 1987). More systematic investigations are however needed to obtain a better quantitative understanding of the influence of illumination wavelength, dosage, cw vs. pulsed laser, etc. for standardized samples. This must go hand-in-hand with more stringent and standardized controls of cell viability, such as well-controlled environmental conditions (in particular temperature, preferably 37 °C), the use of established markers for cell death (Trypan Blue, Propidium Iodide, Annexin V) and/or post-experimental quantifications of cell viability (cell cycle duration, growth rates, or ultimately, single-cell RNA sequencing). Community experience further indicates that single cells are more vulnerable to phototoxicity than entire tissues or living organisms, as larger samples can plausibly dissipate heat more efficiently into their surroundings. While this suggests that a “one size fits all” approach may not always be desirable, an overall better understanding of the involved absorption processes and photochemistry are direly needed to develop clear guidelines for quantitatively assessing and avoiding photodamage or other adverse phototoxic effects in Brillouin microscopy. Similar efforts and approaches as currently being used for fluorescence live cell imaging (Icha et al. 2017) might provide a good starting point.

## Spatial resolution in Brillouin microscopy

The achievable spatial resolution in Brillouin microscopy depends on system parameters such as the optical geometry and the illumination and collection NAs, as well as on intrinsic material properties including the phonon propagation length and the homogeneity of the sample. In some cases, these intrinsic material properties dominate over the optical diffraction limit and therefore impose a fundamental threshold on the achievable and meaningful spatial resolution of a Brillouin imaging system. As a result, there is no straightforward definition of the spatial resolution limit and care must be taken when analyzing highly heterogeneous biological samples, such as cells and tissues.

One of the first aspects that needs to be considered in an attempt of maximizing the *transverse* (lateral) spatial resolution in BM is the inherent spectral broadening caused by the finite numerical aperture (NA) of the illumination and detection lenses (Antonacci et al. 2013). High NA optics introduce a wide range of scattering wavevectors resulting in broadened and distorted Brillouin peaks (Antonacci et al. 2013). An additional non-trivial broadening can also result from the anisotropy in acoustic wave velocity over the probed angles (Elsayad et al. 2020). While the contribution from inherent finite-NA broadening may be accounted for in post-processing steps, the effect of anisotropy poses an additional problem as it requires a priori knowledge of the measured parameters. In orthogonal scattering configuration (*θ* = 90°) the inherent broadening is more pronounced and may lead (especially for high-loss low-storage modulus materials) to a spectral overlap between Brillouin and Rayleigh peaks (which are detected in spontaneous BM), while in backscattering geometry (*θ* = 180°) it is less pronounced and the intrinsic Brillouin line-shape can more readily be recovered upon deconvolution of the instrumental response function (Mattana et al. 2018). From a practical perspective, these broadening effects also have to be seen in the context of typical biological materials where the natural linewidth is in the range of several hundred MHz.

For epi-detection geometry and low NA, in the ideal case of homogeneous and isotropic material, the transverse spatial resolution from a purely optical perspective is dictated by the system’s effective point spread function (PSF) given by the convolution of the illumination and collection PSFs (Antonacci 2017). However, other important aspects must be taken into account when considering (mechanically) heterogeneous materials. Here, both the phonon wavelength and propagation length *l* = *Vτ*, defined by the acoustic velocity *V* and the phonon lifetime *τ*, play an important role. Critically, different cellular biomolecules or compartments (e.g., organelles or lipid membranes) can give rise to an effective phonon propagation length (which is typically of the order of a few microns in liquids (Damzen et al. 2003)) that may be significantly longer than the size of the system’s optical PSF (Prevedel et al. 2019). Conceptually, one can think of this as implying that the acoustic phonons both originate from, and extend into, regions outside of the optical scattering volume (defined by the optical PSF) and thus contribute to high NA measurements. However, given the causal dependence between the real and imaginary parts of a material’s response function, these two length scales can in general not be considered independent. Observation of a reduced transverse resolution for the longitudinal modulus has indeed been reported in correlative Raman (Silvia Caponi and Mattarelli 2020) and fluorescence measurements (Elsayad et al. 2016), the latter two being molecular processes that are subject to the classical diffraction-limited optical resolution. While more rigorous studies are still required to quantify the dependence of the spatial resolution on the material’s parameters, these recent works suggest that the transverse spatial resolution can intricately depend on the nature of the acoustic impedance mismatches at (acoustic) boundaries also in the vicinity of the optical PSF. This would imply a material-specific transverse resolution that depends not only on the phonon velocity but also on the associated phonon attenuation length for finite-NA measurements. Finally, it should be noted that in general the scale of spatial heterogeneities in relation to the characteristic acoustic length scales may also become critical, resulting in finite size/resonance effects and potentially throwing into question the validity of effective medium approximations. While the current opinion is that the transverse resolution can be approximated by the optical resolution, it is still being debated to what extent and in what precise manner regions outside the optical probing volume may contribute. This is a topic currently being actively investigated by the community.

Additional considerations must also be made for the axial spatial resolution in an epi-detection confocal setup, which in conventional diffraction-limited confocal microscopes can be up to a few μm. In a perfectly homogeneous and isotropic material, if the phonon propagation length is shorter than the axial extent of the scattering volume (i.e., *l* < PSF_*z*_) then the natural Brillouin linewidth can be inferred (upon deconvolution of the instrumental response function). On the other hand, if the axial extent of the scattering volume is *shorter* than the phonon propagation length (i.e., PSF_*z*_ < *l*), the detected signal will originate from a damped acoustic wave of a similar nature to that of a truncated Bragg grating. This would result in an additional broadening of the Brillouin peak (following the analogous logic that decreasing the number of grating elements in a dispersion grating negatively affects the spectral resolution). In our opinion, such a broadening should not represent a fundamental obstacle when evaluating the real part of the longitudinal (storage) modulus *M’* as the Brillouin frequency shift would still be obtained with relatively high precision and sensitivity by standard curve fitting. Nevertheless, an accurate measure of the imaginary part of the longitudinal (loss) modulus *M”* would be compromised even in a homogeneous sample since knowledge of the PSF_z_ in relation to the phonon propagation length would be required to correctly extract the latter. Other similar considerations and constraints as for the transverse resolution (see above) can also be expected to apply to the axial resolution in BM.

In summary, a complete rigorous definition of the *mechanical* spatial resolution in Brillouin microscopes requires knowledge of the system’s effective NA, the phonon wavelength and propagation length, as well as the heterogeneity and anisotropy of the sample under investigation. While the pure instrumental broadening due to the system NA can be corrected for by suitable deconvolution, other sources of broadening will be more complex and can impose a fundamental, and in some cases restrictive, limit on the achievable spatial resolution. Ideally, we recommend to assess or measure spatial resolution in a sample-specific and case-by-case basis, and not to refer to purely optical parameters or measurements in other samples or media.

## Quantitative measurements of the longitudinal modulus

The measured Brillouin frequency shift does not only depend on the longitudinal elastic modulus *M* of the specimen but also on its refractive index (RI) and density. Therefore, measurement of the local RI and density for the Brillouin probed volume within a sample is also needed to provide accurate viscoelastic properties of the biological samples. Recently, optical diffraction tomography (ODT) has emerged as a powerful technique for quantifying the local RI distribution in biological samples with a high spatial and temporal resolution by measuring the optical phase delay from various illumination angles (Sung et al. 2009; Abuhattum et al. 2018). A combined BM-ODT setup can extract longitudinal modulus by correlative analysis of the Brillouin frequency shift and RI distribution in the sample. Alternatively, two co-localized Brillouin scattering measurements at different scattering angles can also be used to decouple the local RI distribution from the Brillouin shift within the same probed confocal volume, albeit at currently lower spatial resolution (Fiore et al. 2019).

For the most part in biological samples, the RI is linearly proportional to the dry mass density, i.e., the density of the nonaqueous components (Barer 1952; Popescu et al. 2008). If the sample is considered as a binary mixture of a dry and a fluid fraction, the water content and absolute density of the specimen can also be determined from RI measurements (Schlussler et al. 2018). This approach can provide a reliable estimation of the density for samples containing high water fractions, e.g., zebrafish larvae (which gives a < 4% overestimation of the density). Alternatively, SBS microscopy can assess the mass density via measurements of the Brillouin peak gain, provided knowledge about the RI of the sample (Remer and Bilenca 2016). As the RI of most biological samples lies within the range of *n* ~ 1.35–1.4, while the surrounding aqueous medium typically has *n* = 1.33 (Liu et al. 2016), taking into account the actual RI distribution does not significantly affect the calculation of longitudinal modulus (typically by < 5%). However, it is still necessary to consider the local RI and density distribution of biological samples which may display significant spatial RI heterogeneity due to, e.g., underlying phase transitions or interfaces. An example is lipid droplets in adipocytes consisting of triglycerides and cholesteryl esters, which exhibit a high RI (*n*~1.46) (Yanina et al. 2018) but a low mass density, and would require different relations for deducing the mass density from the measured RI. Since the abundance, and thus the mass density, of lipids can be obtained from the Raman scattering intensity (Oh et al. 2019) for instance, combined Brillouin and Raman microscopy (Palombo et al. 2018; Traverso et al. 2015) may also potentially be applied for correlative studies of the Brillouin shift and the mass density in the case of lipid-rich samples.

## How sample hydration impacts the Brillouin spectrum

Early work has determined that Brillouin spectroscopy can probe the complete elasticity tensor and mechanical anisotropy of protein fibers (Randall et al. 1979; Cusack and Miller 1979; Cusack and Lees 1984; Palombo et al. 2014; Edginton et al. 2016; Koski et al. 2013) and has shown that the longitudinal modulus is a few orders of magnitude higher than the moduli derived from classical biophysical approaches. This discrepancy is thought to arise from the different spatial and temporal scales of the two measurements (at high and low frequencies), as well as from the different types of moduli, i.e., longitudinal modulus vs. shear and Young’s moduli that are more widely used in biophysics and assumed to represent the “stiffness” or “rigidity” (Prevedel et al. 2019; Edginton et al. 2018; Silvia Caponi and Cavalleri 2019; Andriotis et al. 2019). However, the contribution of water to both tissue biomechanics and the Brillouin spectrum is a complicating factor. The former can be explained by pore-elastic models (Mow et al. 1999; Frantz et al. 2010; Margueritat et al. 2019), while the latter is still a subject of debate as to whether in highly hydrated fluids, simulating some aspects of the cell cytoplasm, the frequency shift of the Brillouin peak is determined by modes generated in the water phase (Adichtchev et al. 2019; Wu et al. 2018).

To evaluate the biophysical significance of the information content of the Brillouin spectrum, a recent study has investigated gelatin hydrogels (made of denatured type-I collagen), which are model systems derived from collagen, the most ubiquitous structural protein, and comparable with real tissue samples (Bailey et al. 2019). These gels are homogenous on the phonon wavelength scale, have no hierarchical structure, and their physical properties can be tuned by varying the polymer concentration in order to reproduce the various biological states of matter, ranging from the liquid to the gel and the glassy phase. By reducing the water content, a transition from the liquid phase (low elastic modulus) to the solid phase (high elastic modulus) is revealed in the frequency dispersion and the associated maximum in the linewidth of the Brillouin peak. This liquid–glass transition drives the system towards the solid-like behavior typical of many tissues such as cartilage, tendon, and bone. These results demonstrate that the Brillouin parameters of gelatin hydrogels as model systems for protein networks are dominated by the interaction of solute with the solvent relaxation dynamics and that Brillouin spectroscopy is a unique probe of micromechanics of biological systems with important applications in biology, engineering, physical and medical sciences.

## Approaches to data analysis

Challenges associated with spectral data analysis in Brillouin microscopy have also emerged over the last decade. In most physics and material science applications, one typically does not deal with compromised photon statistics and wavevector-dependent broadening, and as such routine curve fitting approaches with analytical functions are straightforward. In the case of effectively homogeneous regions within biological samples probed with sufficient acquisition times and defined scattering wavevectors, routine deconvolution of the instrumental response function with fitting algorithms based on Lorentzian or Damped Harmonic Oscillator (DHO) functions will in general be valid. However, probing complex spatially heterogeneous biological samples with high spatial and temporal resolution can lead to a number of data analysis problems. These can be roughly categorized as being mainly due to (1) compromised photon statistics and (2) multiple Brillouin sub-peak contributions.

Case (1) can be seen as an inevitable consequence of high-speed BM when employing minimum laser illumination, as is desirable for studying dynamic biological processes and minimizing phototoxic effects. Given that the (spontaneous) Brillouin cross section is inherently small, this problem will exist for BM to different degrees in any live cell imaging application. The problem of assessing the frequency shift of a Brillouin peak amid different noise sources is conceptually the same as that encountered in localization fluorescence microscopy, and similar experimental and data analysis guidelines may thus be followed (e.g., mapping the peaks to correspond to 2–3 pixels on the detector array). In this regard, specific “denoising” approaches based on maximum entropy reconstruction and wavelet analysis have been shown to also be effective for analysis of poor signal-to-noise Brillouin scattering spectra (Xiang et al. 2020), for which practical limits on the extractable information can be derived (Török and Foreman 2019). An alternative approach, which is yet to be demonstrated for BM but likely holds considerable potential, involves denoising by convolution of the spectrum with a Lorentzian function (Farahani et al. 2011).

Case (2) arises when one deals with a spectrum that is a superposition of an unknown number of Brillouin peaks of unknown linewidths. This may arise for several reasons—see section “Spatial resolution in Brillouin microscop*y*” above, but also (Mattana et al. 2018; Prevedel et al. 2019; Mattana et al. 2017). Standard curve fitting with few constraints, especially in the presence of noise, can become challenging or impossible in this scenario. Over the last couple of years a few different approaches, largely taken from fields that face similar challenges, have been implemented to address this limitation. From the realm of hyperspectral imaging, an approach based on Non-negative Matrix Factorization (NMF) has been shown to be very effective in decomposing Brillouin peaks of heterogeneous samples with careful consideration of the relevant length scales (Palombo et al. 2018). An approach based on Spectral Moment Analysis (SMA)—frequently employed for the analysis of Raman spectra—has been shown to be a rapid alternative for plotting an average intensity and frequency shift from multiple peak contributions without recurring to lengthy curve fitting analyses (Fioretto et al. 2019). Finally, an approach routinely applied for the analysis of statistically compromised or complex multi-component time-resolved fluorescence lifetime data, which makes use of spectral phasors, has proved to be beneficial for the analysis of noisy multi-component Brillouin spectra, allowing for virtually real-time analysis and straightforward spectral deconvolution (Elsayad 2019). A common thread in the above approaches is that they require little a priori knowledge of the true Brillouin spectral shape, and in some cases can provide an analytical representation of both the peak positions and linewidths. It remains to be seen if and to what extent these alternative data analysis methods will ultimately prove desirable, with the optimum approach likely also depending on the nature of the dataset and the information one wishes to extract. It is, however, clear that the above described problems of compromised photon statistics and complex spectral profiles and the need for improved and faster data analysis approaches will become more stringent as the community strives to realize the next generation BM instruments with improved temporal, spectral, and/or spatial resolution.

Several additional challenges in regard to data analysis were also identified which include spectral broadening (due to measurement geometry, multiple scattering, etc.) and optimum treatment of the spectra in the vicinity of acoustic boundaries (see section “Spatial resolution in Brillouin microscopy” above) as well as of structures with critical geometric sizes in relation to the relevant acoustic scales. While for the latter the challenges are more formidable and have yet to be rigorously addressed in the context of BM in biological systems, the former is more readily addressable with due diligence, i.e., explicitly accounting for the wavevector-dependent (finite NA) broadening (Antonacci et al. 2013), in addition to that of the instrumental/sample-specific and inhomogeneous broadening. Given that the effective viscosity, as measured by the Brillouin linewidth, is becoming increasingly interesting as a parameter for uncovering the nature of phase transitions, e.g., (Antonacci et al. 2018) (see also discussion of the effect of hydration above) as well as for medical diagnostics and prognostics e.g., (Margueritat et al. 2019), extra attention should be paid to correctly account for all potential broadening effects when analyzing and interpreting complex Brillouin spectra.

## A new standardized unit to report frequency shift

There is significant variability in the literature regarding the quantities and symbols used to present the results of BM studies. For the measured spectral shift of the Brillouin peak (often simply called the “Brillouin shift” or “Brillouin frequency shift”) the symbols ω, Δω, Ω, ΔΩ, *v*, Δ*v*, and *f*, often accompanied by the subscript “B”—for *Brillouin*, or “S” (“AS”)—for *Stokes* (*anti-Stokes*), have all been used to varying degrees. Among the participants of the meeting, it was agreed that when possible the symbol *v*_B_ (nu-B) will be used to describe the Brillouin frequency shift whenever possible, in an attempt to introduce homogeneity in the expanding literature.

A more pressing issue concerns the reported parameters of BM measurements. While *v*_B_ is typically the “raw” measured parameter, which is ideally independent of any additional sample- or instrument-specific assumptions, there are disadvantages in reporting only this value, which include the following: (1) Given its name and dimensionality, it may seem counterintuitive to those unfamiliar with the field that ν_B_ will contain information associated with the mechanical properties. (2) A potentially undesirable aspect of reporting only ν_B_ is that (at least in spontaneous BM) it will scale inversely with the incident laser wavelength. Given the different laser wavelengths used for BM (see above), this inhibits any quick off-the-cuff comparison between values obtained for similar samples in different setups or laboratories.

In light of the above, we propose to report a normalized Brillouin frequency shift which can be used to more readily compare measurements on different systems and to relate the measured mechanics to a common material. This parameter should ideally account for the laser wavelength, yet does not require many additional measurements or assumptions, such that it can be easily and broadly adopted. In particular, the dimensionless quantity ${\overline{\nu}}_B$ (nu-B-bar) is proposed, defined as ${\overline{\nu}}_B={\nu}_B/{\nu}_B^{(W)}-1$. Here *ν*_*B*_ is the measured Brillouin frequency shift in the sample/region of interest and ${\nu}_B^{(W)}$ is the Brillouin frequency shift of distilled water measured using the same setup under the same thermodynamic conditions as those for the sample. ${\overline{\nu}}_B$ will by construction be independent of the laser wavelength used and will to a first approximation correct for the dependence on experimental conditions such as temperature in hydrated materials. Given the sensitivity of the Brillouin spectra to sample hydration (see above) and that water constitutes a significant volume fraction (~70%) of biological samples (as well as typically representing the calibration standard and the “background” signal in live cell studies), expressing the relative deviation of the Brillouin frequency shift from that of water is seen as being both insightful and intuitive. ${\overline{\nu}}_B$ will be dimensionless and can be expected to have a value larger than 0 in most biological samples. It is proposed that the dimensionless parameter ${\overline{\nu}}_B$ can be referred to as the *Brillouin elastic contrast*, to reflect both the means by which it was measured, the information it conveys, and the motivation behind it. Analogously and by the same arguments, a normalized Brillouin linewidth (or *Brillouin viscous contrast*) may also be defined as: ${\overline{\Gamma}}_B={\Gamma}_B/{\Gamma}_B^{(W)}-1$, where Γ_*B*_ and ${\Gamma}_B^{(W)}$ are the measured linewidths (following deconvolution of the instrumental response function, etc.).

Finally, an additional important aspect from a practical perspective in regard to standardization concerns the calibration of spectrometers. This is not always trivial given the very small spectral shifts that are of interest and the associated sensitivity of the instruments. Typically, in imaging spectrometers, the spectra of several calibration standards are taken to suitably scale the dispersion axis of the spectral projection according to an assumed functional dependence of the projection that is calculated for the particular optics in place. Given the sensitivity of the BM spectra to thermodynamic conditions and optical alignment, it is important that such calibration protocols are well described for each study to avoid and be able to account for any systematic errors when comparing measurements in different laboratories or at different time points. While we hope that introduction of a standardized unit (such as the one described above) can help mitigate systematic, as well as to some degree thermodynamic, differences between studies, this should not be seen as relaxing the requirement for frequent calibration/recalibration, especially in the case of imaging spectrometers.

In summary, although there have been many important technical advances over the last decade that have opened up the possibility of using BM to study many diverse live biological samples, it is also clear that there are many challenges still ahead. As instrumentation continues to improve and more diverse biological systems are studied and understood, additional challenges and questions will no doubt emerge. The views expressed in this communication represent the current *status quo* and understood best practices in the use of BM for biomedical studies. It is hoped that, following these suggestions will lead to a reduction of confusion in the approach, terminology, and interpretation of results, which are often found when exploring a nascent field in science.
